# P-1555. Impact of Empiric Treatment Failure on Clinical Outcomes and Healthcare Resource Utilization Among US Females with Uncomplicated Urinary Tract Infections

**DOI:** 10.1093/ofid/ofae631.1722

**Published:** 2025-01-29

**Authors:** Debra L Fromer, Meghan Luck, Malena Mahendran, Rose Chang, Megan Pinaire, Dar Alon, Mei Sheng Duh, Madison T Preib, Jeffrey J Ellis

**Affiliations:** Hackensack University Medical Center / Hackensack Meridian School of Medicine, Hackensack, NJ; GSK; Analysis Group, Inc., Boston, Massachusetts; Analysis Group, Inc., Boston, Massachusetts; Analysis Group, Inc., Boston, Massachusetts; Analysis Group, Inc., Boston, Massachusetts; Analysis Group, Inc., Boston, Massachusetts; GSK; GSK

## Abstract

**Background:**

Management of uncomplicated urinary tract infections (uUTIs) could be impacted by antibiotic (ABX) treatment failure (TF), which may lead to suboptimal outcomes. This study aimed to investigate the impact of TF to empiric oral ABX on clinical outcomes and healthcare resource utilization (HCRU) in female outpatients with uUTI in the United States.

**Figure:**
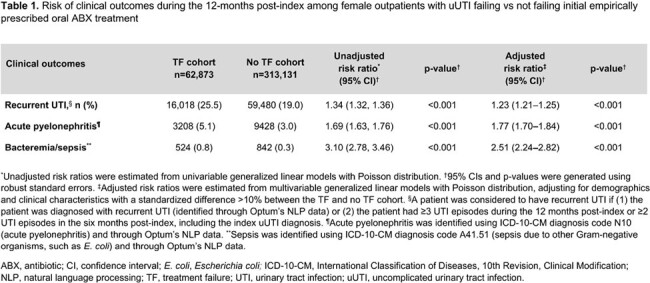

**Methods:**

This study used retrospective data from Optum’s de-identified Electronic Health Record (EHR) dataset (Jan 2017 to Sep 2022). Eligible female outpatients (ambulatory/emergency department [ED] setting) had a UTI diagnosis with no evidence of complicated UTI and ≥ 1 empiric prescription (Rx) for an oral ABX of interest within ±5 days (date of 1st Rx = index date), were ≥ 12 years of age on the index date and had 12 months of EHR activity pre- and post-index. TF was defined as a second oral ABX Rx, administration of intravenous ABX, or an ED visit or inpatient stay with a new primary diagnosis of UTI within 28 days post-index. Risk of clinical outcomes in the 12 months post-index, and rates of all-cause and UTI-related HCRU per 100 person-months during the index uUTI episode and in the 12-months post-index, were compared between patients with vs without TF using multivariable regression models.

**Figure:**
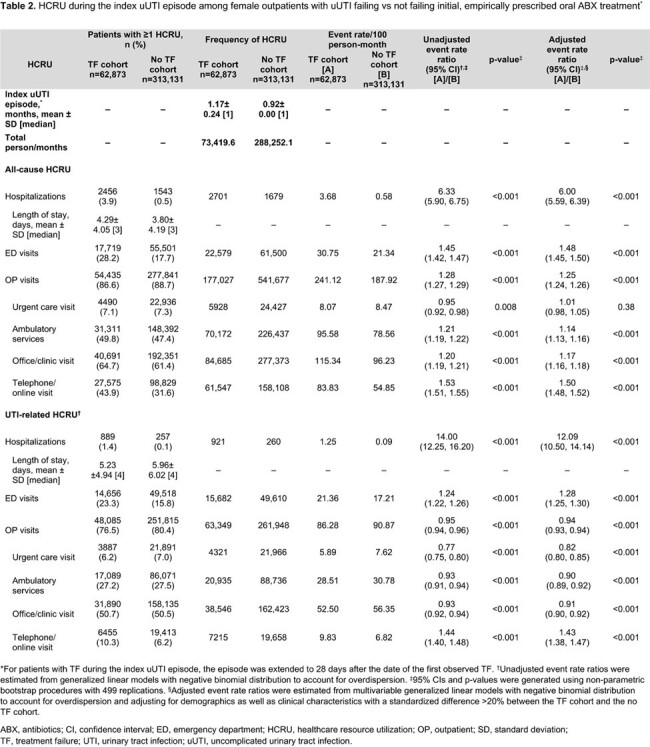

**Results:**

Of 376,004 patients, 62,873 (16.7%) experienced TF. In the 12 months post-index, patients with TF had a higher risk of recurrent UTI (adjusted risk ratio [95% confidence interval (CI)] = 1.23 [1.21–1.25]), acute pyelonephritis (1.77 [1.70–1.84]), and bacteremia/sepsis (2.51 [2.24–2.82]) vs those without TF (all p< 0.001; **Table 1**). Patients with TF also had higher rates of all-cause and UTI-related hospitalizations (adjusted rate ratio [95% CI] = 6.00 [5.59–6.39] and 12.09 [10.50–14.14], respectively) and ED visits (1.48 [1.45–1.50] and 1.28 [1.25–1.30], respectively) during the index uUTI episode (all p< 0.001; **Table 2**). Similar trends were observed in the 12-months post-index (**Table 3**).

**Figure:**
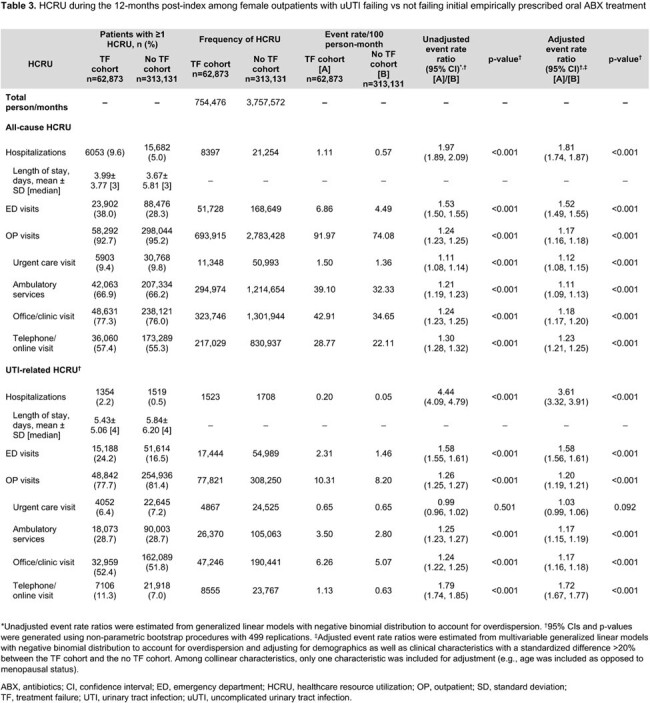

**Conclusion:**

Patients with empiric ABX TF had significantly greater risk of suboptimal clinical outcomes and higher HCRU than patients without TF, underscoring the importance of effective empiric ABX Rx to minimize the short- and long-term burden of TF in uUTI.

**Funding:** GSK study 219500

**Disclosures:**

**Debra L. Fromer, MD**, GSK: Advisor/Consultant|Johnson & Johnson/Janssen Pharmaceuticals: Advisor/Consultant **Meghan Luck, PharmD, BCPS**, GSK: Employee|GSK: Stocks/Bonds (Private Company) **Malena Mahendran, MS**, Analysis Group, Inc.: Employee of Analysis Group, Inc., a consulting company that received funding from GSK to conduct this study **Rose Chang, ScD**, Analysis Group, Inc.: Employee of Analysis Group, Inc., a consulting company that received funding from GSK to conduct this study **Megan Pinaire, MPH**, Analysis Group, Inc.: Employee of Analysis Group, Inc., a consulting company that received funding from GSK to conduct this study **Dar Alon, MS**, Analysis Group, Inc.: Employee of Analysis Group, Inc., a consulting company that received funding from GSK to conduct this study **Mei Sheng Duh, MPH, ScD**, Analysis Group, Inc.: Employee of Analysis Group, Inc., a consulting company that received funding from GSK to conduct this study **Madison T. Preib, MPH**, GSK: Employee|GSK: Stocks/Bonds (Public Company) **Jeffrey J. Ellis, PharmD, MS**, GSK: Employee|GSK: Stocks/Bonds (Public Company)

